# Correspondence

**Published:** 1899-06

**Authors:** 

**Affiliations:** Grand Rapids, Mich.


### CORRESPONDENCE.

GRAND RAPIDS, MICH., June 5, 1899.

EDITOR JOURNAL OF COMPARATIVE MEDICINE, ETC.,
Philadelphia, Pa.

ESTEEMED DOCTOR: Your editorial on the Michigan Veterinary Act before me. We beg to say that the Grand Rapids school did and does now advocate a three-years' course of study; but we do not propose to be coerced into assisting the other American schools to drive our American boys, who are rich in brains and poor in purse, into Canada, where they can, at the present time, get a certificate in two five-months' sessions that will bring them an income equalling that of the man holding a diploma from any American college. Those certificates from the Toronto school are recognized in the majority of the States of the Union, and so long as they are —Toronto remaining a two-years' school—just so long will the Grand Rapids school remain a two-years' school. The Toronto school has grown rich at the expense of American shortsightedness. The majority of American laymen are not educated up to a standard sufficiently high to judge between the ability of a man holding a diploma from a two-years' school and that of a better trained man.

For these reasons we claim that the time is not yet ripe for us to extend our curriculum to three years. The recent Michigan Veterinary Act was framed by the Michigan State Veterinary Association, and is a very poor, weak thing, indeed; in fact, it is not worthy of a place on our statute books. It is no better than no law at all, and time will demonstrate this fact. There is not one man, I venture to say, belonging to the Michigan State Veterinary Association who is a graduate of a three-years' school; then why are they setting up such a howl and cry for higher education? We are free to admit that the average man holding a certificate from a two-years' school is a poor stick. And why? Simply because he is stuffed full of theory. The teachers are theorists, the school is theoretical, and clinical work is unknown.

In the Grand Rapids school all this is different. The teachers are all practical men, and clinical work is of daily occurrence. Veterinary legislation in Michigan has been manipulated by the State Veterinary Society for a quarter of a century, and now we presume they are proud of their great efforts (?). However, from the low grade of legislation that they have been able to secure it is evident that our legislative body does not take much stock in the average veterinarian.

Yours truly,

GRAND RAPIDS VETERINARY COLLEGE.

